# Neuropathic symptoms after sleeve gastrectomy: two cases illustrating diagnostic pitfalls and discordant nerve conduction findings – a case report

**DOI:** 10.1097/RC9.0000000000000924

**Published:** 2026-09-01

**Authors:** Ahmad E. Al-Mulla, Abdulla E. Sultan, Obaid Al-Harbi, Nour A. Jibara

**Affiliations:** a Kuwait Board of General Surgery (KBGS), Fellowship Minimal Invasive, Bariatric and Endoscopy (Brazil), Fellowship American College of Surgeons (FACS), Department of Surgery, Farwaniya Hospital, Ministry of Health Kuwait (MOH), Farwaniya, Kuwait; b Department of Surgery, Farwaniya Hospital, Ministry of Health Kuwait, Farwaniya, Kuwait; c Department of Surgery, Farwaniya Hospital, Ministry of Health Kuwait, Farwaniya, Kuwait

**Keywords:** bariatric surgery, malnutrition, peripheral neuropathy, sleeve gastrectomy, thiamine deficiency

## Abstract

**Introduction:**

Peripheral neuropathy is a recognized but often underappreciated complication after sleeve gastrectomy, particularly in patients with rapid weight loss, recurrent vomiting, poor oral intake, and inadequate adherence to postoperative supplementation.

**Presentation of case:**

We describe two young women who developed neuropathic symptoms within 3–4 months of laparoscopic sleeve gastrectomy. Both experienced substantial early weight loss, poor oral intake, recurrent vomiting or regurgitation, and poor adherence to prescribed supplements. Serum vitamin B12 and thiamine levels were elevated in both cases. In the first case, nerve conduction studies demonstrated mild axonal sensorimotor polyneuropathy. In the second, symptoms were neuropathic in character, but lower-limb electrophysiological studies were normal, and interpretation was complicated by spinal imaging abnormalities and interrupted treatment adherence.

**Clinical discussion:**

These cases underscore the limitations of routine serum vitamin measurements in excluding clinically relevant nutritional neuropathy and highlight the importance of considering alternative neurological or musculoskeletal explanations while initiating prompt empirical treatment in high-risk patients.

**Conclusion:**

Early nutritional reassessment and empirical vitamin replacement should be considered in symptomatic patients after sleeve gastrectomy, particularly when poor intake and rapid postoperative weight loss are present.

## Introduction

Sleeve gastrectomy is currently the most commonly performed bariatric operation worldwide and is often viewed as metabolically and nutritionally safer than malabsorptive procedures[1]. However, neurological complications can still occur and may be under-recognized when early postoperative warning signs such as recurrent vomiting, regurgitation, reduced intake, and rapid weight loss are attributed solely to expected recovery after surgery[2]. Peripheral neuropathy has been linked to deficiencies of thiamine, vitamin B6, vitamin B12, copper, folate, and other micronutrients; importantly, routine serum measurements may not accurately reflect functional intracellular deficiency, which can complicate diagnosis and delay treatment^[^2,3^]^.

HIGHLIGHTS

- Sleeve gastrectomy may precede neuropathic symptoms despite normal vitamin levels.
- Two cases expose diagnostic pitfalls in suspected post-bariatric neuropathy.
- Nerve conduction studies were discordant with the clinical presentation.
- Functional deficiency remained plausible despite reassuring serum markers.
- Multidisciplinary assessment strengthened the diagnostic interpretation.

We present two patients who developed neuropathic symptoms within 3–4 months of sleeve gastrectomy despite elevated serum vitamin levels and discordant electrophysiological findings. These cases highlight an uncommon early postoperative presentation, the diagnostic limitations of routine laboratory testing and nerve conduction studies, and the need for early multidisciplinary assessment. This case report is presented in accordance with the SCARE criteria.

### Case presentation 1

A 23-year-old woman was admitted to a tertiary hospital 4 months after laparoscopic sleeve gastrectomy. Her preoperative weight was 104 kg (body mass index [BMI] 38.2 kg/m^2^), and she had lost approximately 20 kg in the preceding 3 months. She presented with progressive paresthesia, burning pain in both soles, and bilateral lower-limb sensory symptoms that had worsened over several days. There was no history of trauma, recent febrile illness, diarrhea, or toxin exposure.

The postoperative course had been notable for poor oral intake, recurrent vomiting, and non-adherence to prescribed vitamin supplementation. On examination, she had distal sensory impairment in both lower limbs, painful ambulation, and mild right-sided weakness graded 4/5 on the Medical Research Council scale. Knee reflexes were preserved, and there were no upper motor neuron signs.

Routine laboratory evaluation, including inflammatory markers and basic metabolic testing, was largely unremarkable, except for elevated serum levels of vitamin B12, thiamine, and vitamin B6. Nerve conduction studies demonstrated findings consistent with mild axonal sensorimotor polyneuropathy, supporting a peripheral neuropathic process despite the absence of a classic biochemical deficiency profile.

The differential diagnosis included nutritional neuropathy, inflammatory neuropathy such as Guillain–Barré syndrome, metabolic or endocrine causes, toxin- or medication-related neuropathy, and spinal pathology. Nutritional neuropathy was favored because of rapid weight loss, vomiting, poor intake, poor supplement adherence, bilateral distal sensory symptoms, preserved reflexes, no upper motor neuron signs, unremarkable inflammatory and metabolic test results, and nerve conduction findings consistent with mild axonal sensorimotor polyneuropathy.

She was reviewed jointly by neurology, clinical nutrition, psychiatry, and the bariatric surgery team. Management focused on nutritional optimization and symptom control, including empirical intravenous thiamine, multivitamins, and trace elements, together with counseling regarding oral intake and adherence to long-term supplementation.

Her symptoms improved noticeably within 5 days of treatment. She was discharged ambulatory, with a plan for continued thiamine therapy, oral micronutrient supplementation, dietary follow-up, and outpatient neurological review. At follow-up 2 weeks later, she reported further symptomatic improvement and better adherence to nutritional advice.

### Case presentation 2

A 20-year-old woman with hypothyroidism presented to a tertiary hospital with bilateral lower-limb burning pain and tingling, 4 months after laparoscopic sleeve gastrectomy. Her preoperative weight was 98 kg (BMI 38 kg/m^2^), and she had lost 20 kg over 4 months. Because of persistent regurgitation and reflux symptoms, she had discontinued levothyroxine, several prescribed postoperative medications, and vitamin supplements; she also reported persistently poor oral intake.

Her symptoms had begun approximately 1 month earlier and were predominantly nocturnal. She did not report objective weakness, sphincter symptoms, or clear gait impairment, but the pain was severe enough to disturb sleep and daily activities.

Laboratory testing, including thyroid function tests, was within normal limits except for elevated levels of serum vitamin B12, thiamine, and vitamin B6. This again raised the possibility that standard blood measurements did not adequately capture functional nutritional status.

Previous spinal imaging reportedly showed a mild L5–S1 disc protrusion with foraminal narrowing, introducing diagnostic uncertainty about a possible radicular contribution to her symptoms. She had also received intermittent analgesic and vitamin injections at another facility before admission.

The differential diagnosis included nutritional neuropathy, L5–S1 radiculopathy, hypothyroid-related neuropathy, medication interruption, functional pain amplification, and local musculoskeletal or soft-tissue causes. Nutritional neuropathy remained clinically plausible given rapid weight loss, poor intake, regurgitation, and poor supplement adherence; however, normal thyroid function, normal neurological examination, normal lower-limb nerve conduction studies, and spinal imaging abnormalities prevented definitive attribution to nutritional neuropathy alone.

She underwent a multidisciplinary assessment by the clinical nutrition, bariatric surgery, neurology, spine surgery, and psychiatry teams, and a structured nutritional rehabilitation plan was initiated.

Neurological examination remained normal, and repeat nerve conduction studies did not show evidence of lower-limb peripheral neuropathy, despite the neuropathic quality of the patient’s symptoms. This discordance complicated interpretation and broadened the differential diagnosis.

She received empiric intravenous multivitamins and trace elements; however, she left against medical advice after 7 days, limiting both the completion of the inpatient workup and the formal assessment of treatment response.

One month later, she was re-admitted with recurrent symptoms, bilateral lower-limb swelling, and an infected ulcer after a traditional therapy-related skin injury, which introduced an additional confounding source of pain and functional limitation.

She was treated with antibiotics, wound care, and repeated vitamin therapy. At follow-up, her symptoms had improved alongside better adherence to nutritional advice and supplementation, although the exact contribution of each intervention could not be determined.

The key clinical features, investigations, management strategies, and outcomes of both cases are summarized in Table 1.

**Table 1:** Comparative summary of the clinical features, investigations, management, and outcomes of the two cases.

Parameter	Case 1	Case 2
Age	23 years	20 years
Comorbidities	None	Hypothyroidism (previously on levothyroxine 50 micrograms)
Time from surgery to presentation	4 months after SG	4 months after SG
Weight loss	20 kg	20 kg
Symptoms	Burning feet, paraesthesia, gait impairment	Tingling/burning, no objective weakness; recurrence complicated by a burn from traditional cauterization-based home therapy
Risk factors	Recurrent vomiting, poor intake, non-adherence	Reflux, regurgitation, non-adherence
TSH	–	TSH 1.36 uIU/L; reference range: 0.38–5.33 uIU/mL (normal)
Vitamin B12 level	>1118 pmol/L (elevated)	1476 pmol/L (elevated)
Thiamine (vitamin B1) level	322 nmol/L (above normal)	265.2 nmol/L (above normal)
Vitamin B6 level	979 nmol/L (above normal)	560.7 nmol/L (above normal)
Other micronutrients	Within normal limits	Within normal limits
Imaging	–	MRI: Mild L5–S1 disc protrusion with muscle spasm.
Nerve conduction studies	Axonal sensorimotor polyneuropathy	Upper limb: carpal tunnel syndrome; lower limb: no electrophysiological evidence of lower-limb neuropathy.
Thiamine regimen	100 mg IV TDS for 7 days; 100 mg IV once daily for 7 days; oral thiamine for 1 month	300 mg IV TDS for 7 days
Other management	IV vitamins B6, A, D, E; zinc; copper; nutritional support	IV multivitamins; zinc; copper; wound care after burn from traditional cauterization-based home therapy; IV antibiotic (piperacillin–tazobactam 4.5 g TDS)

## Discussion

Neuropathic complications are reported less frequently after sleeve gastrectomy than after malabsorptive bariatric procedures, yet they remain clinically relevant, particularly in patients with rapid postoperative weight loss, vomiting or regurgitation, poor oral intake, and poor adherence to prescribed supplementation^[^2–5^]^. Both patients in this report had several of these recognized risk factors, supporting a nutritional mechanism even though their serum vitamin levels did not show classic deficiency patterns.

Compared with previously published reports, our cases share recognized postoperative risk factors, including rapid weight loss, vomiting or regurgitation, poor oral intake, and inconsistent supplementation^[^2–5^]^. Similar to earlier reports of polyneuropathy after sleeve gastrectomy, the first patient developed distal sensory symptoms with electrophysiological evidence of axonal sensorimotor polyneuropathy^[^3,6^]^. However, our report differs by highlighting elevated serum vitamin levels despite clinically suspected nutritional neuropathy, and discordance between neuropathic symptoms and normal lower-limb nerve conduction studies in the second patient. Unlike reports centered on overt deficiency or established neurological syndromes, these cases emphasize early recognition, empirical treatment, and multidisciplinary postoperative management rather than operative revision or further surgical intervention^[^2,4,7,8^]^.

The principal lesson from these cases is that clinically important neuropathic symptoms may follow sleeve gastrectomy even when routine serum vitamin concentrations are normal or elevated^[^6,7^]^. Functional biomarkers such as methylmalonic acid, homocysteine, erythrocyte transketolase activity, or more detailed micronutrient assays were not available in our setting, so the diagnosis remained primarily clinical rather than biochemically confirmed. Diagnostic confidence was greater in the first patient, whose examination and electrophysiology were consistent with mild axonal sensorimotor polyneuropathy. In the second patient, normal lower-limb studies, spinal imaging abnormalities, treatment non-adherence, and later soft-tissue injury limited causal inference and underscored the complexity of postoperative neuropathic presentations. Taken together, these cases support early nutritional reassessment and empirical vitamin replacement while alternative neurological and musculoskeletal causes are evaluated in parallel^[^2,4,8^]^.

From a practical clinical perspective, these cases emphasize that new paresthesia, burning pain, gait difficulty, or distal sensory symptoms after sleeve gastrectomy should prompt early nutritional reassessment, even when routine serum vitamin levels are normal or elevated. Recurrent vomiting or regurgitation, rapid weight loss, reduced oral intake, and poor adherence to supplementation should be regarded as warning signs rather than expected postoperative symptoms. These factors may also influence postoperative management by identifying patients who require closer surveillance, early dietetic review, a low threshold for empirical thiamine and multivitamin replacement, and timely multidisciplinary input from bariatric surgery, nutrition, neurology, and mental health teams. Early recognition and coordinated management may reduce diagnostic delay, prevent progression, and improve functional recovery in similar patients.

Similarly, rare surgical case reports from resource-limited settings show how transparent reporting of diagnostic uncertainty, multidisciplinary decision-making, and pragmatic management can inform clinical practice[9].

## Limitations

This report has several limitations. Functional biomarkers such as methylmalonic acid and homocysteine were unavailable, and serum vitamin concentrations may not correlate reliably with intracellular or tissue-level deficiency. In the second case, interpretation was further limited by spinal imaging abnormalities, interrupted adherence to therapy, outside interventions, and later skin injury, each of which may have contributed to symptoms. Accordingly, these observations are best interpreted as clinically informative and hypothesis-generating rather than definitive proof of a single mechanism.

## Conclusion

Neuropathic symptoms may emerge within months of sleeve gastrectomy, particularly in patients with rapid weight loss, prolonged vomiting or regurgitation, poor oral intake, and inconsistent use of postoperative supplements. Early recognition, nutritional reassessment, empirical vitamin replacement, and simultaneous evaluation of alternative causes are essential to reduce morbidity and avoid diagnostic delay.

## Data Availability

Not applicable.

## Patient perspective

Both patients described their symptoms as distressing and functionally limiting, particularly because the pain interfered with mobility, sleep, and routine daily activities. They reported that nutritional counseling, vitamin replacement, and structured follow-up improved their understanding of the condition and reinforced the importance of adherence to long-term supplementation after bariatric surgery.
